# Matrix Development for the Detection of Phosphorylated Amyloid-β Peptides by MALDI-TOF-MS

**DOI:** 10.1021/jasms.2c00270

**Published:** 2023-01-27

**Authors:** Thomas Liepold, Hans-Wolfgang Klafki, Sathish Kumar, Jochen Walter, Oliver Wirths, Jens Wiltfang, Olaf Jahn

**Affiliations:** † Neuroproteomics Group, Department of Molecular Neurobiology, Max Planck Institute for Multidisciplinary Sciences, 37075 Goettingen, Germany; ‡ Department of Psychiatry and Psychotherapy, University Medical Center Goettingen, Georg-August-University, 37075 Goettingen, Germany; § Department of Neurology, University of Bonn, 53127 Bonn, Germany

## Abstract

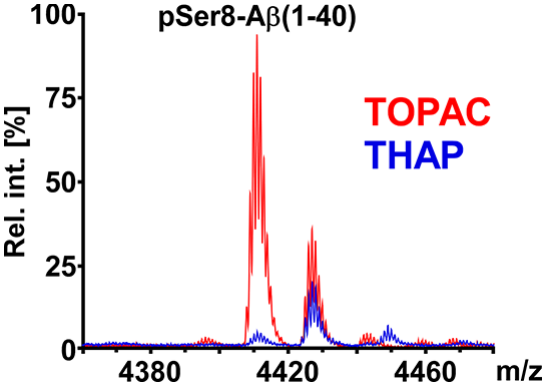

Amyloid-β (Aβ) peptides, including post-translationally modified variants thereof, are believed to play a key role in the onset and progression of Alzheimer’s disease. Suggested modified Aβ species with potential disease relevance include Aβ peptides phosphorylated at serine in position eight (pSer8-Aβ) or 26 (pSer26-Aβ). However, the published studies on those Aβ peptides essentially relied on antibody-based approaches. Thus, complementary analyses by mass spectrometry, as shown for other modified Aβ variants, will be necessary not only to unambiguously verify the existence of phosphorylated Aβ species in brain samples but also to reveal their exact identity as to phosphorylation sites and potential terminal truncations. With the aim of providing a novel tool for addressing this still-unresolved issue, we developed a customized matrix formulation, referred to as TOPAC, that allows for improved detection of synthetic phosphorylated Aβ species by matrix-assisted laser desorption/ionization time-of-flight mass spectrometry. When TOPAC was compared with standard matrices, we observed higher signal intensities but minimal methionine oxidation and phosphate loss for intact pSer8-Aβ(1–40) and pSer26-Aβ(1–40). Similarly, TOPAC also improved the mass spectrometric detection and sequencing of the proteolytic cleavage products pSer8-Aβ(1–16) and pSer26-Aβ(17–28). We expect that TOPAC will facilitate future efforts to detect and characterize endogenous phosphorylated Aβ species in biological samples and that it may also find its use in phospho-proteomic approaches apart from applications in the Aβ field.

Amyloid-β (Aβ) peptides are believed to play a key role in the onset and progression of Alzheimer’s disease (AD). The characteristic neuritic plaques representing one of the classical neuropathological hallmarks in the brains of AD patients are composed of aggregated and highly insoluble Aβ peptides.^1^ Soluble Aβ peptides are generated during normal cellular metabolism by consecutive proteolytic cleavages of the amyloid-beta precursor protein (APP) by β- and γ-secretases and can be found under physiological conditions in biological fluids, such as cerebrospinal fluid (CSF) and blood plasma (reviewed in ref (2)). Oligomeric forms of Aβ were shown to have synapto- and neurotoxic properties and may thus trigger the synapse and neuron loss observed in AD.^3^ Apart from the “canonical” Aβ(1–40) and Aβ(1–42) variants, the highly insoluble neuritic amyloid plaques also contain modified Aβ peptides, of which the N-terminally truncated forms such as Aβ(4–40/42)^1^ and pyroglutamate-modified AβN3pE-40/42^4^ are particularly abundant.^5,6^ Other reported post-translational modifications of Aβ include isomerization and racemization of aspartic acid residues,^7,8^ nitration of tyrosine,^9^ phosphorylation of serine,^10,11^ and citrullination of arginine.^12^

Among the Aβ peptides reported to be post-translationally modified at central amino acid residues, the species that gained particular interest are phosphorylated at serine in position eight (pSer8-Aβ) or 26 (pSer26-Aβ), of which the former has been studied in more detail.^10,11,13^ The phosphorylated species pSer8-Aβ and pSer26-Aβ appear to have a higher propensity to form aggregates with increased neurotoxicity, similar to what has been proposed for the N-terminally truncated Aβ species.^14,15^ Published studies on the occurrence of pSer8-Aβ and pSer26-Aβ in tissue samples from human brain or transgenic mice relied on detection by selective antibodies directed against these phosphorylated Aβ variants.^11,13,16−20^ However, even the most selective phosphorylation-specific antibodies may show some minor cross-reactivity to the nonphosphorylated versions of their targets, which can lead to inconclusive results when those are present in large excess. Furthermore, a recent study using co-immunocapture on an electrochemical sensor indicated that 5–10% of total Aβ is phosphorylated in CSF but without revealing the exact identity of the phosphorylated species.^21^ Thus, it will be essential to corroborate these studies by the unequivocal verification of the proposed Aβ species in biological samples, ideally by mass spectrometry (MS). Despite their potential pathological relevance, the available mass spectrometric evidence for the occurrence of endogenous pSer8-Aβ and pSer26-Aβ in biological samples is ambiguous at best (see ref (22) for a recent review), underscoring the need for improved methods for the detection of these specific Aβ species by MS.

As the combination of immunoprecipitation with matrix-assisted laser desorption/ionization time-of-flight (MALDI-TOF) MS is a widely used method in the Aβ field, in general, and forms the basis of a prominent blood biomarker assay for cerebral Aβ accumulation, in particular,^23−25^ we sought to address the technical challenges of detecting phosphorylated Aβ peptides by MALDI-TOF-MS. Relevant for the mass spectrometric analysis of all intact Aβ species, these challenges include the commonly known limited “flyability” of Aβ peptides, likely related to the characteristic high hydrophobicity of their C-termini, and the susceptibility for oxidation at methionine in position 35 during sample preparation, which further decreases the detectability of the target peptide due to signal splitting. Specific to phosphorylated Aβ peptides, detection of the intact species suffers from the typical loss of meta-phosphoric (HPO_3_, −80 Da) or phosphoric acid (H_3_PO_4_, −98 Da) under mass spectrometric conditions, at least when analyzed by MALDI-TOF-MS in reflector mode.^26^ Nevertheless, data acquisition in the reflector mode should be the method of choice for the characterization of (novel) post-translationally modified Aβ species by MALDI-TOF-MS, as it provides the required high mass accuracy needed for unequivocal assignments, in contrast to data acquisition in the linear mode. To set the groundwork for future studies aiming for a mass spectrometric verification of phosphorylated Aβ peptides in brain samples from transgenic mice or AD cases, we present here the development of a customized matrix formulation, referred to as TOPAC. Starting from 2′,4′,6′-trihydroxyacetophenone with diammonium citrate (THAP/DAC),^27^ a matrix preparation widely used for the analysis of phosphorylated peptides in positive ion MALDI-TOF-MS, we show how selected additives result in a matrix that allows for the detection of synthetic pSer8-Aβ(1–40) and pSer26-Aβ(1–40) with high signal intensities, while minimizing Met oxidation and phosphate loss. We suggest that the TOPAC matrix formulation, which includes as additives the nonionic detergent *n*-octyl-β-d-glucopyranoside (OGP), phosphoric acid (PA), and the commonly used peptide matrix compound α-cyano-4-hydroxycinnamic acid (CHCA),^28^ will facilitate the characterization of phosphorylated Aβ species by MALDI-TOF-MS at the level of both the intact Aβ peptides and their proteolytic cleavage products.

## Experimental Section

### Synthetic Aβ Peptides

Synthetic Aβ(1–40), pSer8-Aβ(1–40), and pSer26-Aβ(1–40) were obtained from Peptide Specialty Laboratories (Heidelberg, Germany).

### Matrix Preparations

All chemicals were from Sigma-Aldrich/Merck; α-cyano-4-hydroxycinnamic acid (CHCA, catalog number 70990), 2′,4′,6′-trihydroxyacetophenone (THAP, #91928), diammonium citrate (DAC, #09831), *n*-octyl-β-d-glucopyranoside (OGP, #494459), phosphoric acid 85% (PA, #30417). The following matrix preparations were used: CHCA, saturated solution in 50% acetonitrile (ACN); THAP/DAC, 10 mg/mL THAP dissolved in 50% ACN/0.5% DAC; THAP/DAC+OGP, 10 mg/mL THAP dissolved in 50% ACN/0.5% DAC/0.1% OGP. For the preparation of the final matrix formulation containing all additives (referred to as TOPAC), 0.6 μL of PA (0.5% final concentration) and 1.0 μL of saturated CHCA (see above) were added to 100 μL of the THAP/DAC+OGP matrix. For dried-droplet preparations, 1.0 μL of the respective matrix solution was mixed with 1.0 μL of Aβ peptide dilution (10 ng/μL in 0.1% trifluoracetic acid, TFA), deposited on a Ground Steel Target Plate (Bruker, #8280784), and dried under ambient conditions. Calibrant spots were prepared in the same way with 1.0 μL of a 1:1 mixture of the peptide standards Peptide Calibration Standard II (Bruker, #8222570) and PepMix2 (LaserBio Labs (see https://laserbiolabs.com/), #C102), providing a calibrant range of up to 6000 mass-to-charge (*m*/*z*).

### Proteolytic Digestion

Aβ peptides (1 μg) were incubated with endoproteinase Lys-C (Roche, #11047825001) in 50 mM ammonium bicarbonate/10% ACN with an enzyme-to-substrate ratio of 1:5. After overnight incubation (24 h) at 37 °C, digestion was stopped with TFA (0.5–1% final concentration), and 1 μL aliquots were directly subjected to MALDI-TOF-MS without further cleanup.

### Mass Spectrometry and Data Analysis

Mass spectrometric analysis of intact Aβ peptides was essentially performed as described recently.^29^ Briefly, positively charged ions in the *m*/*z* range of 600–6000 were recorded in the reflector mode of an MALDI-TOF/TOF mass spectrometer of the Bruker Ultraflex series (Ultraflex I operated under flexControl 3.3 or UltrafleXtreme operated under flexControl 3.4). A total of 1000 (Ultraflex I) or 5000 (UltrafleXtreme) spectra per sample were recorded from different spot positions while keeping the laser fluence constant to minimize inter- and intraexperimental variation of signal intensities. Semiquantitative comparison of signal intensities was limited to THAP-based matrices, for which the laser attenuator settings had to be approximately doubled compared to spectra acquisition from CHCA matrix. Images of MALDI target plate spots were derived from screen shots taken from the graphical user interface of flexControl 3.3/3.4. The software flexAnalysis 3.3/3.4 (Bruker) was used to calibrate and annotate mass spectra and to determine intensities and signal-to-noise ratios of signals.

The software flexImaging 4.1 (Bruker) was used to map the analyte incorporation into matrix spots with spatial resolution. Spectra were acquired from a raster of 100 μm spacing. False-colored images of signals from Aβ peptides of interest were generated using a mass window of ±3 Da around the calculated monoisotopic mass and overlaid onto the corresponding matrix image scanned at a resolution of 1200 dpi with an Epson 1680 Pro scanner.

Peak lists of fragment ion mass spectra of proteolytic Aβ peptide cleavage products were searched against the Swiss-Prot primary sequence database restricted to the taxonomy *Homo sapiens* (release 2022–03, 20 398 entries) using the MASCOT Software 2.3 (Matrix Science). For MS/MS ion searches, phosphorylation of Ser/Thr residues was specified as variable modifications, and mass tolerances were set to 100 ppm for precursor ions and 0.7 Da for fragment ions. Enzyme was specified as “none” to account for the fact that Aβ peptides are released from amyloid-beta precursor protein (APP, Swiss-Prot accession P05067) by β- and γ-secretase cleavage.

## Results and Discussion

**Figure 1:**
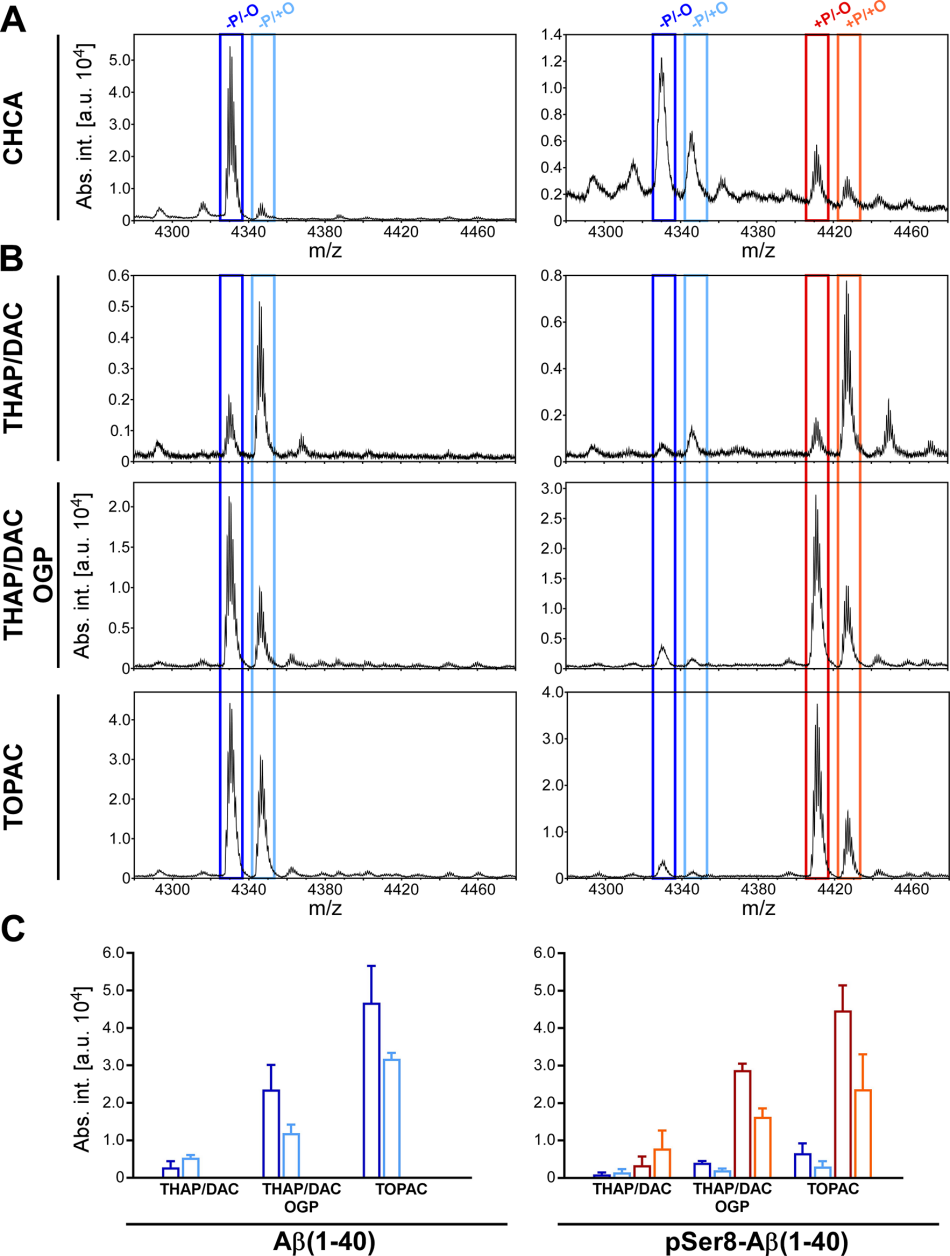
Detection of intact Aβ(1–40) and pSer8-Aβ(1–40) by MALDI-TOF-MS. Mass spectra of Aβ(1–40) (left column) and pSer8-Aβ(1–40) (right column) were acquired from CHCA (A) or from THAP with different matrix additives (B). Within the THAP-based matrices, data acquisition conditions were kept constant to allow for the semiquantitative assessment of signal intensities (mean of *n* = 3 ± standard deviation) shown in (C). Specific signals in the mass spectra are highlighted by colored framing as follows: dark blue, nonphosphorylated Aβ without Met oxidation (-P/-O); light blue, nonphosphorylated Aβ with Met oxidation (-P/+O); red, phosphorylated Aβ without Met oxidation (+P/-O); orange, phosphorylated Aβ with Met oxidation (+P/+O). The same color code is used for the bar graphs in (C).

As a starting point for our matrix development, we first compared the mass spectra of Aβ(1–40) and pSer8-Aβ(1–40) when acquired either from CHCA (commonly used for peptides in general) or from THAP/DAC (commonly used for phospho-peptides). While CHCA produced high signal intensities for Aβ(1–40) in reflector mode already at low laser fluence, most of pSer8-Aβ(1–40) (Figure 1A) was found to undergo loss of meta-phosphoric acid (−80 Da) under these conditions. This was not surprising, as CHCA is known to be a “hot” matrix that comes with extensive in-source fragmentation and postsource decay of fragile peptide ions. Notably, the extent of undesirable Met oxidation for Aβ(1–40) was negligible, and also for pSer8-Aβ(1–40), the nonoxidized species was still the more abundant signal (Figure 1A). The opposite scenario was found when THAP/DAC was used as the matrix. Here, the phosphate loss from pSer8-Aβ(1–40) was negligible, but the Met-oxidized species were the dominant signals for both Aβ(1–40) and pSer8-Aβ(1–40) (Figure 1B,C). Obviously, the oxidation happened during matrix crystallization, thereby compromising the detection sensitivity for the actual target signals. Moreover, despite the higher laser fluence that was required to be able to record spectra from THAP/DAC, absolute signal intensities were considerably lower than with CHCA, e.g., about an order of magnitude in the case of Aβ(1–40) (Figure 1B,C). Taken together, we reasoned that the THAP/DAC matrix needs to be improved with regard to a reduction of its oxidative properties and an enhancement of its ionization efficiency to be applicable for the detection of phosphorylated Aβ peptides.

As the first and apparently most effective step of a series of improvements, we applied the nonionic detergent OGP as matrix additive. We and others have shown that the application of the MALDI-compatible OGP at all levels of gel-based proteomic workflows, i.e., in-gel digestion, extraction of proteolytic peptides from the gel, and matrix preparation, led to improvements in recovery and MALDI-MS response especially of large, hydrophobic peptides.^30−34^ In particular, the addition of OGP to CHCA matrix not only increased signal intensities for proteolytic peptides as originally shown by Cohen & Chait^30^ but also prevented oxidation at Met residues due to a protective effect of OGP as first observed by Nordhoff and colleagues.^31^ We thus hypothesized that the detection of pSer8-Aβ(1–40) may benefit from the addition of OGP to the THAP/DAC matrix. From the tested concentrations in the range of 0–1% (Figure S1A), we selected 0.1% OGP as additive and found that the corresponding matrix preparation (referred to as THAP/DAC+OGP) led to a considerable increase in signal intensity without promoting phosphate loss (Figure 1B). More importantly, the addition of OGP switched the relative intensities of the split signals for the oxidized and reduced species, respectively, so that the actual target signals representing nonoxidized Aβ(1–40) and pSer8-Aβ(1–40) were now most abundant (Figure 1B,C). Thus, both proposed effects of OGP—enhancement of the MALDI-MS-response and protection from Met oxidation—positively affected the detection of phosphorylated Aβ peptides. Given that OGP is composed of an aliphatic part and a carbohydrate ring, we speculate that it is this particular chemical structure that is capable of both improving polypeptide solubility and preventing excessive oxidation at Met residues.

Next, we set out to fine-tune the properties of our matrix preparation with the aim of increasing the signal intensities for phosphorylated Aβ peptides further without compromising the beneficial effects on the prevention of phosphate loss and Met oxidation, respectively. Stimulated by the work of Kjellstrom & Jensen^35^ to use PA as a matrix additive for 2,5-dihydroxybenzoic acid (DHB), we added PA to THAP/DAC+OGP to test if it leads to the previously observed enhancement of phospho-peptide ion signals in this matrix as well. This effect was mainly attributed to a passivation of iron ions on the surface of the ground steel MALDI target plate, diminishing metal binding of phospho-peptides.^36^ Moreover, we assumed that an additional spiking of the THAP/DAC+OGP matrix with CHCA may further enhance signal intensities as long as it is applied carefully in small amounts to keep phosphate loss minimal. We tested additive concentrations in the range of 0–2% for PA (Figure S1A′) and 0–5% for CHCA (Figure S1A″) and chose to supplement the THAP/DAC+OGP matrix with 0.5% PA and 1% CHCA, which resulted in the final TOPAC formulation. Indeed, TOPAC allowed the detection of pSer8-Aβ(1–40) with the highest signal intensities observed throughout our matrix development, minimal phosphate loss, and an optimized ratio between the nonoxidized target species and the undesired oxidized species of more than 2:1 (Figure 1B,C). Notably, the matrix additives in TOPAC did not lead to any additional interfering matrix cluster signals in mass spectra of vehicle only (0.1% TFA) when compared with THAP/DAC (Figure S1B). To exclude that heterogeneous analyte distributions such as the so-called “coffee-ring effect”^37^ may have affected our observations during matrix development, we assessed the incorporation of a 1:1 mixture of Aβ(1–40) and pSer8-Aβ(1–40) into THAP/DAC and TOPAC by MALDI imaging and found a comparable analyte distribution in both matrices (Figure S1C).

**Figure 2:**
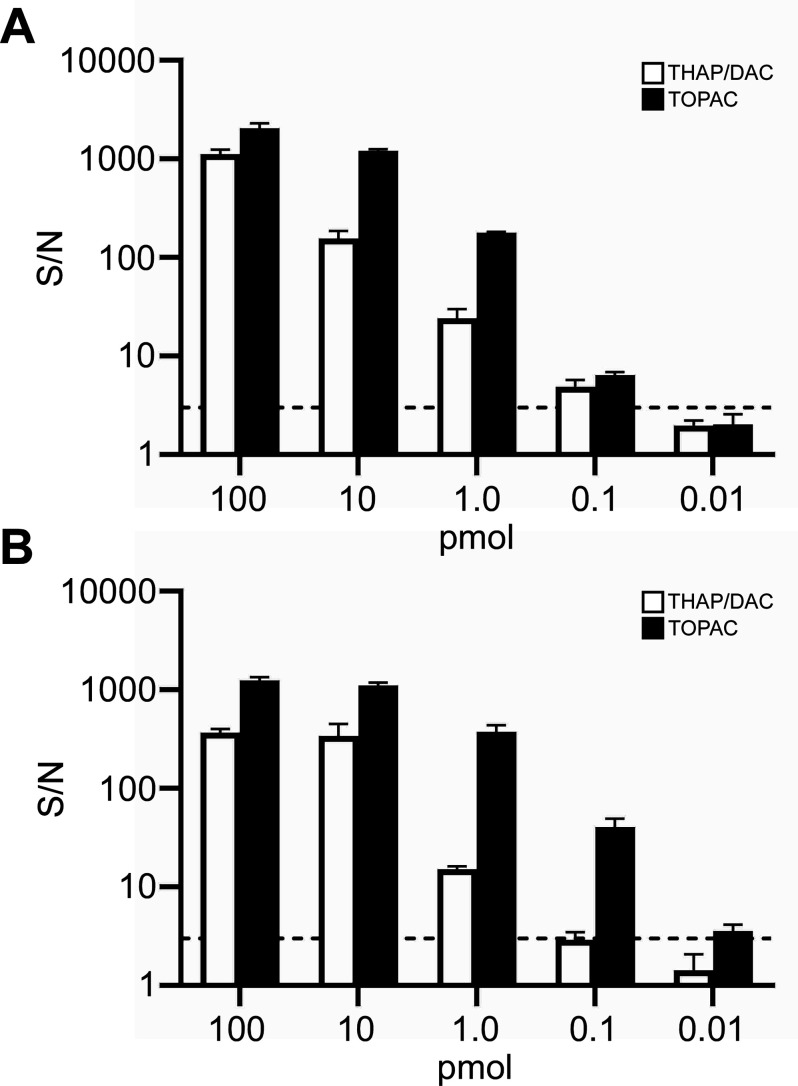
S/N determined from dilution series of Aβ peptides. These ratios were derived from signals for Aβ(1–40) (A) and pSer8-Aβ(1–40) (B) using THAP/DAC (open bars) or TOPAC (filled bars) as matrices. S/N for the respective amount on target (in pmol) are plotted on a logarithmic scale with standard deviations from three independent experiments. S/N = 3 (marked by stippled line) was considered as detection limit. Note that 10 fmol pSer8-Aβ(1–40) is still detectable with the TOPAC matrix.

Since the semiquantitative assessment of absolute signal intensities implied a gain in sensitivity for TOPAC over THAP/DAC (Figure 1, Figure S2), we next investigated the detection limits of phosphorylated Aβ peptides for both matrices. Following earlier work from Schiller and colleagues on the relationship between signal-to-noise ratio (S/N) and sample amount on target,^38,39^ we analyzed dilution series of Aβ(1–40) and pSer8-Aβ(1–40) in the range of 0.01–100 pmol on target and determined the S/N of the respective signals, for which we defined a threshold of S/N = 3 as the detection limit. As shown in Figure 2, the TOPAC matrix consistently produced higher S/N in the upper-to-medium concentration range for both Aβ peptides under study. For pSer8-Aβ(1–40), but not for Aβ(1–40), the same held true for the lower concentration range, resulting in a detection limit of approximately 100 fmol on target for pSer8-Aβ(1–40) in THAP/DAC, whereas it was found to be approximately 10 fmol on target in TOPAC (Figure 2B).

Finally, to confirm that our findings are extendable to other phosphorylated Aβ species, we also tested our matrix development with synthetic pSer26-Aβ(1–40). Overall, we obtained similar results as to high phospho-peptide ion signals, solely the extent of Met oxidation as a hardly controllable artifactual event was found to be more variable (Figure S2A–C). Similarly, pSer26-Aβ(1–40) was also detectable down to an amount of approximately 10 fmol on-target, though with less appreciable dependency on the TOPAC matrix (Figure S2D).

Next, we moved from the analysis of individual Aβ peptides to mixtures of unmodified and phosphorylated Aβ peptides to assess the selectivity of the enhanced MS response seen with TOPAC. When 1:1 mixtures of Aβ(1–40) and pSer8-Aβ(1–40) or Aβ(1–40) and pSer26-Aβ(1–40) were analyzed with THAP/DAC and TOPAC, respectively, a considerable boost in signal intensity for the phosphorylated Aβ peptides was confirmed for TOPAC (Figure S3A,B). Interestingly, calculation of the ratios between the signal intensities of phosphorylated and unmodified Aβ peptides indicated that the signal enhancement is somewhat selective for the phosphorylated Aβ peptides, at least in the case of pSer26-Aβ(1–40) (Figure S3C).

After successful detection of intact phosphorylated Aβ species, the subsequent mapping of the phosphorylation site(s) may be desired, which typically involves proteolytic digestion followed by mass spectrometric sequencing of the cleavage products. We thus tested whether the benefits of the TOPAC matrix for the detection of intact phosphorylated Aβ peptides also held true for the analysis of their proteolytic cleavage products. For this purpose, we first digested pSer8-Aβ(1–40) or pSer26-Aβ(1–40) by endoproteinase Lys-C. Similar to our findings with the intact Aβ peptides, we observed an overall increase in MS response with TOPAC in comparison to THAP/DAC (Figure S4A). Notably, the signal enhancement for pSer8-Aβ(1–16) and pSer26-Aβ(17–28) seen with TOPAC did not come at the cost of a higher phosphate loss, as its extent was found to be similar for both matrices, in the range of 5–7% of the target signal (Figure S4A). To assess a potentially selective signal enhancement of phosphorylated Aβ cleavage products, we analyzed proteolytic peptides derived from a Lys-C digest of a 1:1:1 mixture of Aβ(1–40), pSer8-Aβ(1–40), and pSer26-Aβ(1–40) (Figure S4B). However, while an overall increase in MS response with TOPAC was confirmed, calculation of the ratios between phosphorylated Aβ cleavage products [pSer8-Aβ(1–16), pSer26-Aβ(17–28)] and their unmodified counterparts [Aβ(1–16), Aβ(17–28)] did not reveal such selectivity (Figure S4C).

**Figure 3:**
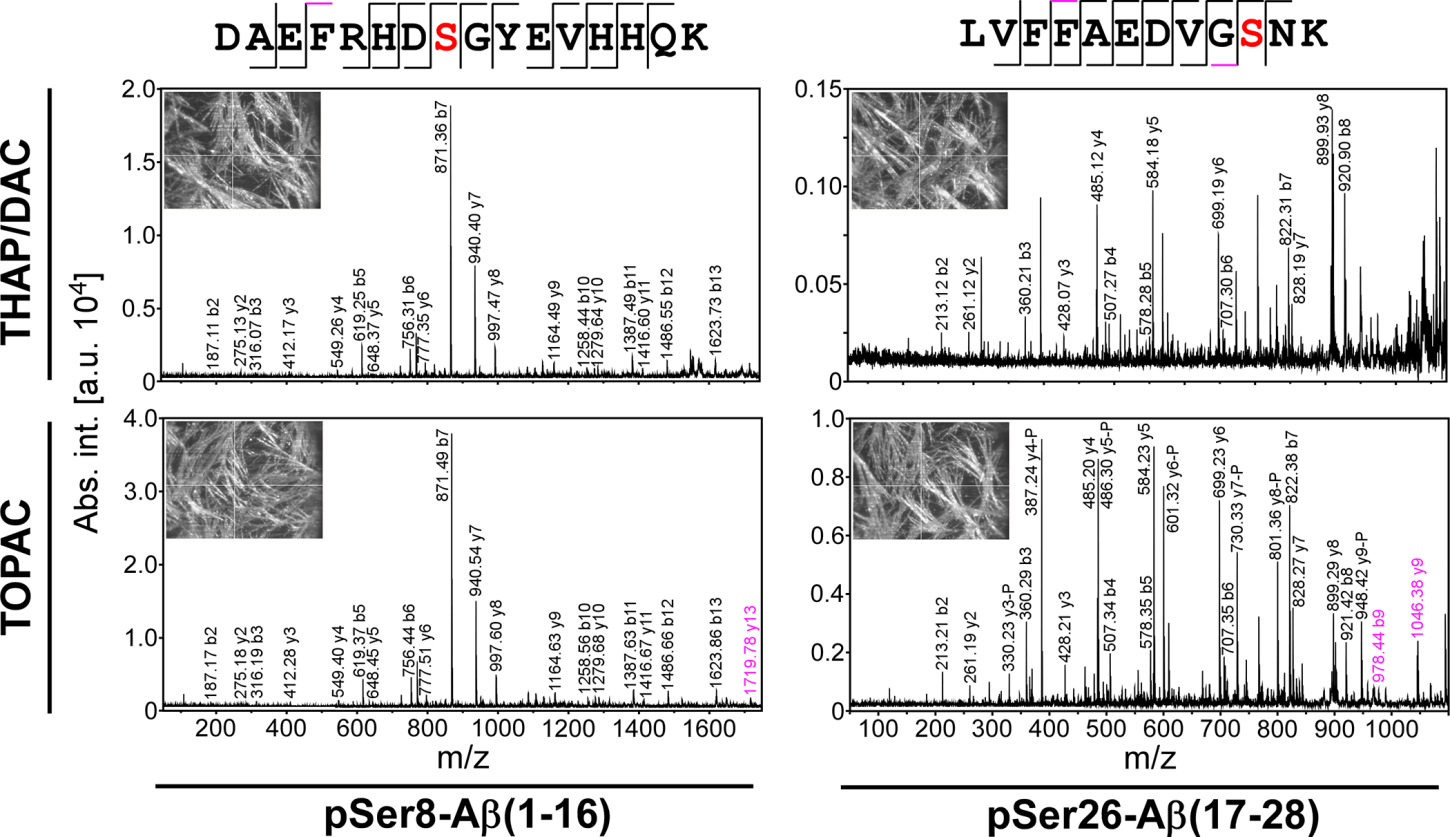
Sequencing of phosphorylated Aβ cleavage products by MALDI-TOF/TOF-MS/MS. Fragment ion mass spectra of pSer8-Aβ(1–16) (left column) and pSer8-Aβ(17–28) (right column) were acquired from THAP/DAC (upper row) and TOPAC (lower row). Only b- and y-ion series are labeled for the sake of clarity. For pSer8-Aβ(1–16), phosphorylation was clearly assigned to Ser-8 (marked in red) on the basis of conclusive N- and C-terminal ion series. The high abundance of the N-terminal b7-ion was in agreement with the preferential cleavage of peptide bonds C-terminal of Asp under the conditions of mass spectrometric peptide sequencing. Similarly, for pSer26-Aβ(17–28), phosphorylation was clearly assigned to Ser-26 (marked in red). Here, the y-ion series was more abundant in agreement with charge localization at the C-terminal Lys residue and was accompanied by a y-ion series showing loss of phosphoric acid (−98 Da, only annotated and marked by -P in the fragment ion mass spectrum from TOPAC matrix). Additional fragment ions detected exclusively from TOPAC matrix are marked in magenta. The images of the MALDI target plate spots (insets) show a virtually identical crystallization behavior of both matrices. This indicates that the matrix additives of the TOPAC formulation apparently did not alter the crystallization behavior of THAP/DAC, which may be of relevance when automated data acquisition is desired.

Finally, we examined the fragmentation behavior of phosphorylated peptides and sequenced pSer8-Aβ(1–16) and pSer26-Aβ(17–28) by MALDI-TOF/TOF-MS/MS. For both peptides, nearly complete fragment ion series were obtained for pSer8-Aβ(1–16) and pSer26-Aβ(17–28) from both THAP/DAC and TOPAC, allowing the assignment of the phosphorylation to Ser-8 and Ser-26, respectively (Figure 3). Importantly, the fragment ion mass spectra from TOPAC showed higher overall intensities with similar increases as observed before for the precursor ions, probably due to the spiking with CHCA. This led to an improved signal quality and thereby to the annotation of additional fragment ions. For an unbiased assessment of the quality of the fragment ion mass spectra, peak lists were submitted to a database search via Mascot. For the mass spectra acquired from THAP/DAC, scores of 56 (38 in an independent digest replicate) for pSer8-Aβ(1–16) and 40 (34) for pSer26-Aβ(17–28) were obtained for the identification of amyloid-beta precursor protein (APP), while the scores were found to be 67 (60) for pSer8-Aβ(1–16) and 67 (58) for pSer26-Aβ(17–28) when TOPAC was used for data acquisition. Given that the thresholds for identity were in the score range of 52–54, we suggest that the higher spectral quality obtained with TOPAC was decisive for unambiguous protein identification of APP and for the correct assignment of the phosphorylation sites in pSer8-Aβ(1–16) and pSer26-Aβ(17–28), respectively.

## Conclusion

The characterization of phosphorylated Aβ peptides is challenging at all levels, from sample preparation to mass spectrometric analysis. In general, the phosphoester bond may be hydrolyzed during the harsh conditions that are required for solubilizing aggregated Aβ. More specifically, the commonly used procedures for monomerization of Aβ peptides under highly basic conditions can lead to beta-elimination of the phosphate group.^40^ To address the technical challenges coming with the analysis of intact phosphorylated Aβ peptides by MALDI-TOF-MS we chose to enhance the MS response via matrix additives, rather than selective enrichment of phospho-peptides. The latter is less straightforward, as it typically involves separate steps such as immobilized metal affinity chromatography.^41^ We have developed TOPAC as a customized matrix formulation that allows for the sensitive detection of intact phosphorylated Aβ species. We further propose it as a valuable tool for future studies aiming for the mass spectrometric verification of phosphorylated Aβ peptides in brain samples and for revealing their exact molecular identity. While this manuscript was under revision, another study reported the detection of synthetic pSer8-Aβ(1–42) by MALDI-TOF-MS with a detection limit of 1 pmol.^42^ Although this result may not be directly comparable with our findings on pSer8-Aβ(1–40), we suggest that the sensitivity for the detection of phosphorylated Aβ peptides can be increased by 1–2 orders of magnitude when TOPAC is used as the matrix. Future applications to biological samples, likely in combination with enrichment by immunoprecipitation, will have to show whether the gain in sensitivity achievable with TOPAC will be sufficient for the detection of the low-abundant phosphorylated Aβ species in tissue lysates or body fluids.

The identification of specific phosphorylated Aβ species from brain samples requires the unambiguous assignment of the potential phosphorylation sites in Aβ peptides, which is a particular challenge, as Ser-8 and Tyr-10 have to be distinguished. The TOPAC matrix may be helpful to address this challenge, as we have shown its improved performance also for the mass spectrometric detection and sequencing of proteolytic cleavage products of phosphorylated Aβ peptides. Although not further explored here, we expect that the TOPAC matrix will be broadly applicable for the analysis of phospho-peptides apart from proteolytic Aβ cleavage products.

## Supporting Information Available

Figure S1 (Development and characterization of the TOPAC matrix), Figure S2 (Detection of intact pSer26-Aβ(1–40) by MALDI-TOF-MS), Figure S3 (Detection of intact phosphorylated Aβ peptides from mixtures with Aβ(1–40)), and Figure S4 (Detection of phosphorylated Aβ cleavage products by MALDI-TOF-MS) (PDF)
