# Supplementary material for: Matrix Development for the Detection of Phosphorylated Amyloid-β Peptides by MALDI-TOF-MS
Source: J Am Soc Mass Spectrom. 2023 Jan 27;34(3):505–12. doi: 10.1021/jasms.2c00270 (PMC9983008; doi:10.1021/jasms.2c00270)
Supplement: Supplementary file 1 — js2c00270_si_001.pdf [file js2c00270_si_001.pdf]

Supporting Information

Matrix development for the detection of phosphorylated Amyloid-β peptides by MALDI-TOF-MS

Thomas Liepold, Hans-Wolfgang Klafki, Sathish Kumar, Jochen Walter, Oliver Wirths, Jens Wiltfang, and Olaf Jahn

Content

Supplementary Figures S1-S4

Supplementary Figure S1

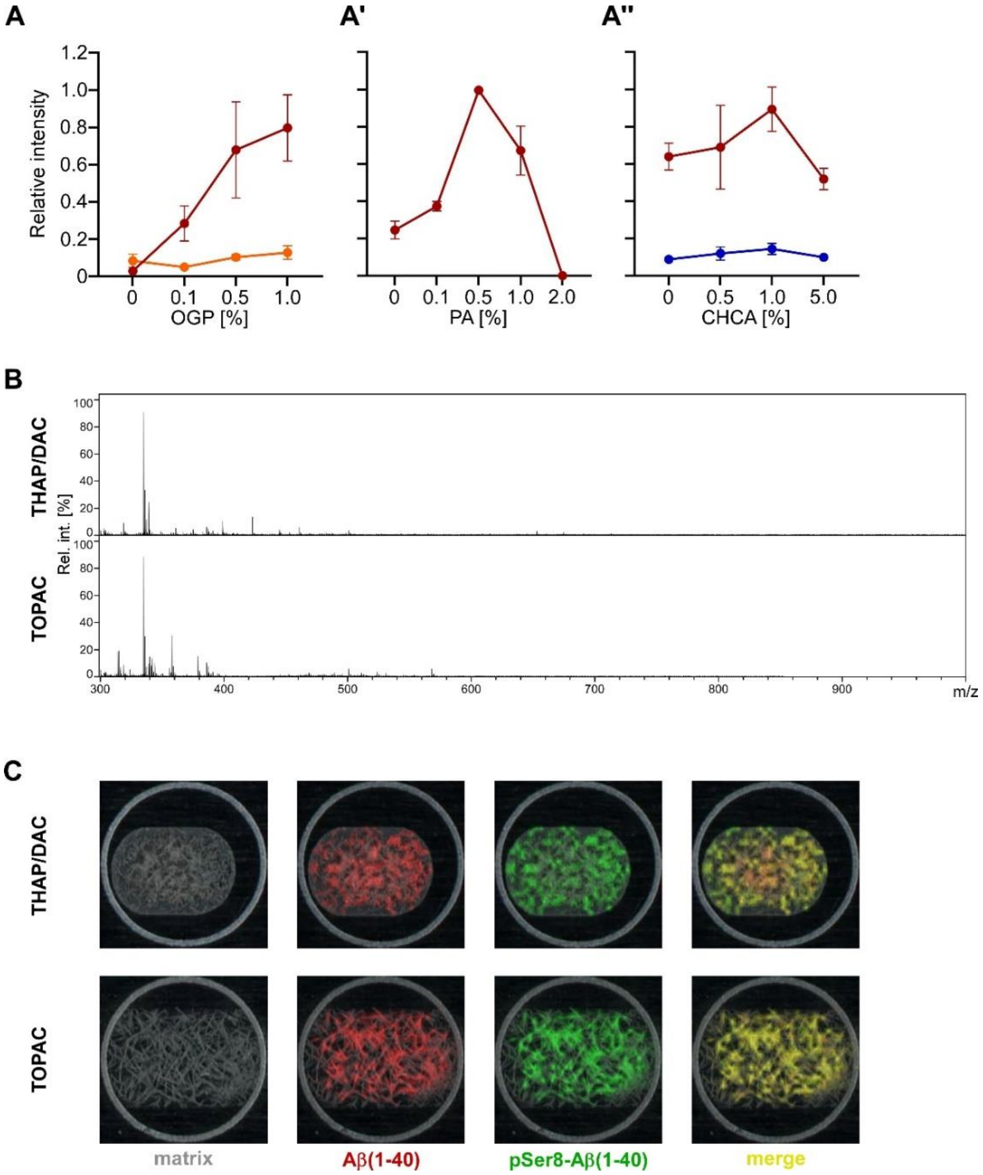

Figure S1. Development and characterization of the TOPAC matrix. Starting from THAP/DAC, OGP was tested as the first matrix additive in the concentration range of 0-1% (A). For further matrix development, 0.1% OGP was selected as the concentration of choice based on the ratio of the MS response for pSer8-Aβ(1-40) (red) and its undesired Met-oxidized version (orange), and on the reproducibility of the measurements (see large error bars for pSer8-Aβ(1-40) with 0.5 and 1% OGP). PA was added to THAP/DAC+OGP in the concentration range of 0-2% (A'). A clear optimum of the MS response for pSer8-Aβ(1-40) was found with the addition of 0.5% PA. Note that the addition of 2% PA virtually precludes any measurement due to matrix crystallization problems. Finally, the MS response for pSer8-Aβ(1-40) was increased further by the addition CHCA (A''). From the concentrations tested (0-5%), 1% CHCA was selected as the concentration of choice based on the ratio of the MS response for pSer8-Aβ(1-40) (red) and its counterpart that has undergone phosphate loss (blue). Error bars show standard deviations from three independent experiments. As shown in (B), the matrix additives in TOPAC (lower panel) did not lead to additional interfering matrix cluster signals in mass spectra of vehicle only (0.1% TFA as used for intact Aβ peptides) when compared with THAP/DAC (upper panel). MALDI imaging of a 1:1 mixture of Aβ(1-40) and pSer8-Aβ(1-40) (C) revealed a homogenous incorporation of the analytes into THAP/DAC (upper row) and TOPAC (lower row). MS response for Aβ(1-40) ($[M+H]^+$ = 4328.16 ± 3) and pSer8-Aβ(1-40) ($[M+H]^+$ = 4408.12 ± 3) was false-colored in red and green, respectively, and overlayed onto the matrix image. Merging of the Aβ(1-40) and the pSer8-Aβ(1-40) channel leads to a yellow color indicative of an equal distribution of both analytes.

Supplementary Figure S2

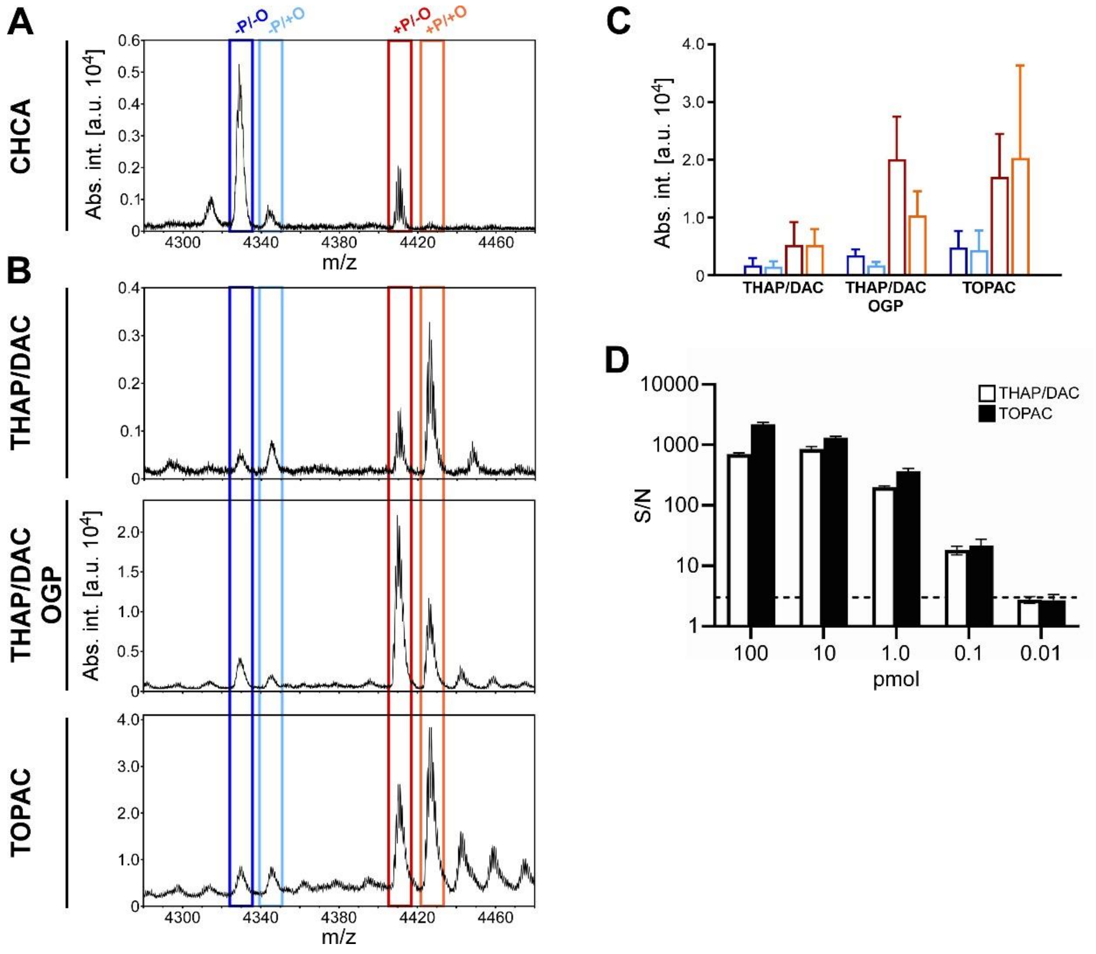

Figure S2. Detection of intact pSer26-Aβ(1-40) by MALDI-TOF-MS. Mass spectra of pSer26-Aβ(1-40) were acquired from CHCA (A) or from THAP with different matrix additives (B). Within the THAP-based matrices, data acquisition conditions were kept constant to allow for the semi-quantitative assessment of signal intensities (mean of n = 3 ± standard deviation) shown in (C). Specific signals in the mass spectra are highlighted by colored framing as follows: dark blue, non-phosphorylated Aβ without Met oxidation (-P/-O); light blue, non-phosphorylated Aβ with Met oxidation (-P/+O); red, phosphorylated Aβ without Met oxidation (+P/-O); orange, phosphorylated Aβ with Met oxidation (+P/+O). The same color code is used for the bar graphs in (C). Signal-to-noise ratios (S/N) in (D) were determined from dilution series of pSer26-Aβ(1-40) using THAP/DAC (open bars) and TOPAC (filled bars) as matrices. S/N for the respective amount on target (in pmol) are plotted on a logarithmic scale with standard deviations from three independent experiments. S/N = 3 (marked by stippled line) was considered as detection limit.

Supplementary Figure S3

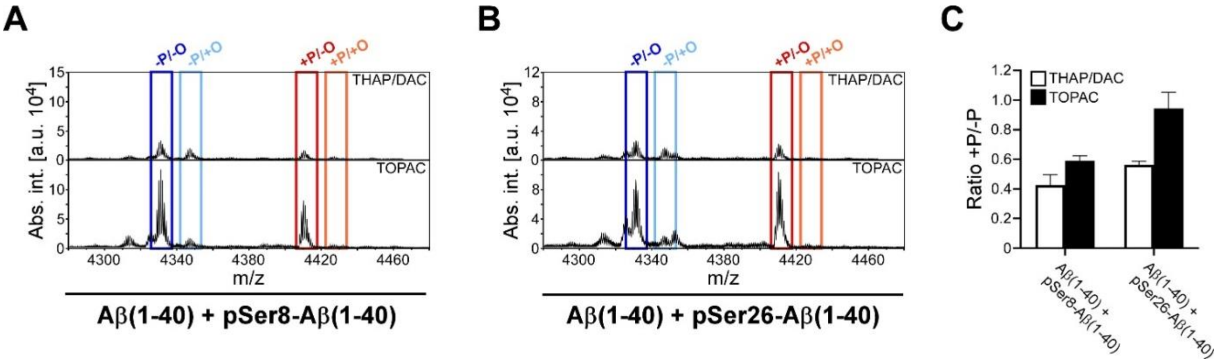

Figure S3. Detection of intact phosphorylated Aβ peptides from mixtures with Aβ(1-40). Mass spectra of 1:1 mixtures pSer8-Aβ(1-40) + Aβ(1-40) (A) and pSer26-Aβ(1-40) + Aβ(1-40) (B) were acquired from THAP/DAC (upper spectra) or TOPAC (lower spectra). Specific signals in the mass spectra are highlighted by colored framing as follows: dark blue, non-phosphorylated Aβ without Met oxidation (-P/-O); light blue, non-phosphorylated Aβ with Met oxidation (-P/+O); red, phos-phorylated Aβ without Met oxidation (+P/-O); orange, phosphorylated Aβ with Met oxidation (+P/+O). The ratios between phosphorylated and unmodified Aβ peptides plotted in (C) were calculated from absolute intensity (int) values as follows: ($int_{+P/-O}$ + $int_{+P/+O}$) / ($int_{-P/-O}$ + $int_{-P/+O}$). Error bars indicate standard deviations from three independent experiments.

Supplementary Figure S4

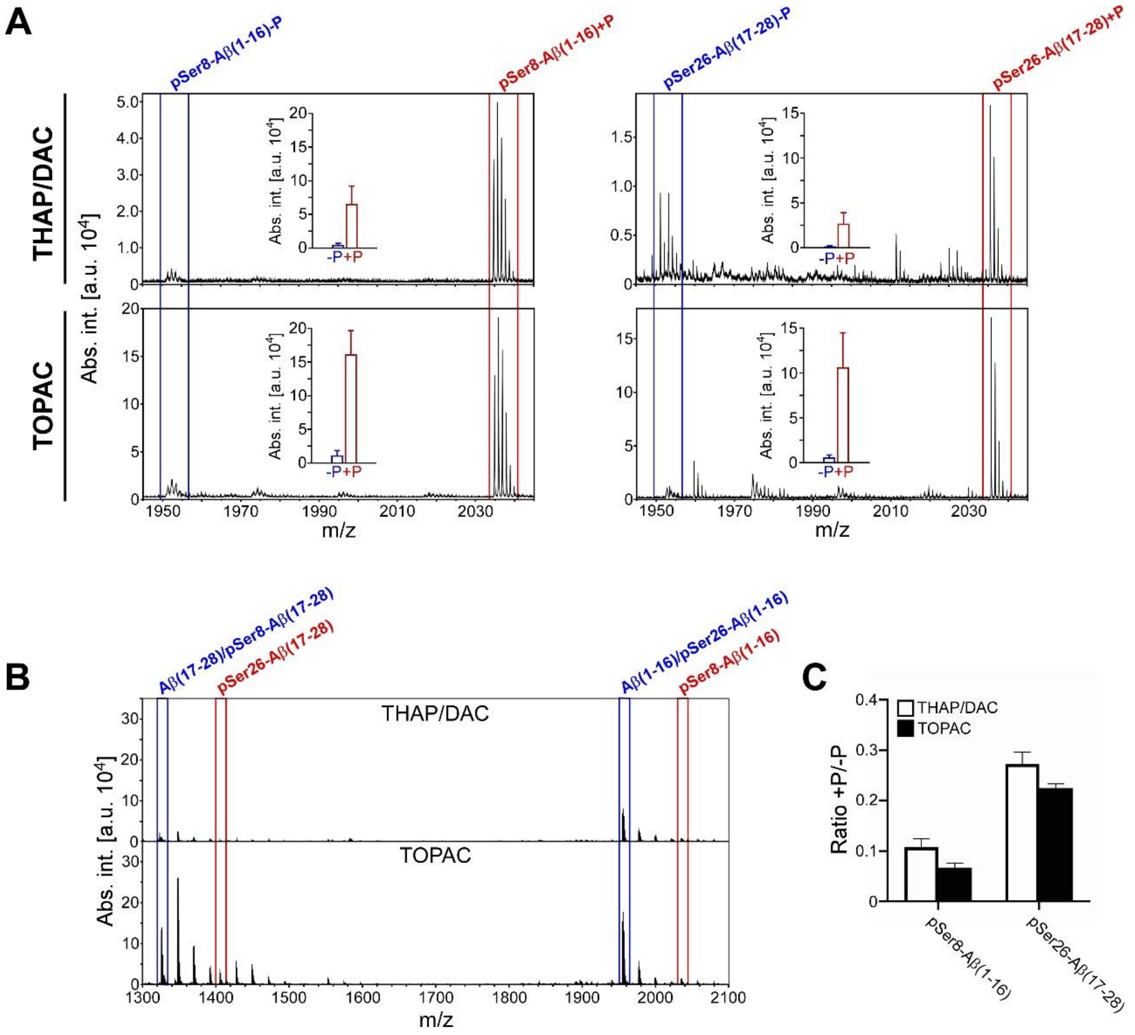

Figure S4. Detection of phosphorylated Aβ cleavage products by MALDI-TOF-MS. Mass spectra of pSer8-Aβ(1-16) (A, left column) and pSer26-Aβ(17-28) (A, right column) were acquired from THAP/DAC (upper row) or TOPAC (lower row). In (A), pSer8-Aβ(1-40) and pSer26-Aβ(1-40) were digested individually to facilitate the semi-quantitative assessment of phosphate loss as shown in the insets. In (B), a 1:1:1 mixture of Aβ(1-40), pSer8-Aβ(1-40), and pSer26-Aβ(1-40) was digested to facilitate comparisons between phosphorylated Aβ cleavage products and their unmodified counterparts as shown in (C). Note that adduct signal series are visible in the mass spectra due to the presence of sodium cations, the source of which was traced back to the Lys-C formulation. Non-phosphorylated Aβ cleavage products are highlighted in dark blue; phosphorylated Aβ cleavage products in red. The ratios between phosphorylated and unmodified Aβ peptides plotted in (C) were calculated from absolute intensity values of the respective signals. Error bars indicate standard deviations from three independent experiments.
